# Protease profile of normal and neoplastic mast cells in the human bone marrow with special emphasis on systemic mastocytosis

**DOI:** 10.1007/s00418-021-01964-3

**Published:** 2021-01-25

**Authors:** Dmitri Atiakshin, Igor Buchwalow, Peter Horny, Markus Tiemann

**Affiliations:** 1 Peoples’ Friendship University of Russia, Moscow, Russia; 2 Research Institute of Experimental Biology and Medicine, Voronezh N. N. Burdenko State Medical University, Voronezh, Russia; 3 Institute of Hematopathology, Fangdieckstr. 75a, 22547 Hamburg, Germany; 4 Institute of Pathology, Ludwig-Maximilians University, Munich, Germany

**Keywords:** Mast cells, Bone marrow, Mastocytosis, Tryptase, Chymase, Carboxypeptidases

## Abstract

Mast cells (MC) are immune cells that produce a variety of mediators, such as proteases, that are important in the body’s immune responses. MC proteases have pronounced multifunctionality and in many respects determine the biological characteristics of the organ-specific MC population. Although, increased numbers of MC are one of the objective mastocytosis signs, a detailed assessment of the proteases biogenesis and excretion mechanisms in the bone marrow (BM) has not yet been carried out. Here, we performed an analysis of the expression of proteases in patients with various forms of systemic mastocytosis. We presented data on intracellular protease co-localization in human BM MCs and discussed their implication in secretory pathways of MCs in the development of the disease. Systemic mastocytosis, depending on the course, is featured by the formation of definite profiles of specific proteases in various forms of atypical mast cells. Intragranular accumulation of tryptase, chymase and carboxypeptidases in the hypochromic phenotype of atypical mast cells is characterized. Characterization of MC proteases expression during mastocytosis can be used to refine the MC classification, help in a prognosis, and increase the effectiveness of targeted therapy.

## Introduction

Mastocytosis, a type of mast cells (MC) disease, is caused by the accumulation of functionally defective MCs and MC precursors. When MCs undergo degranulation, the substances that are released can cause several symptoms that can vary over time and can range in intensity from mild to severe. The histochemical features of biosynthesis products of MCs made it possible for Paul Erlich to determine MC on micropreparations more than 130 years ago (Ehrlich 1877, 1878). The arsenal of synthesized products in MCs is very diverse, including glycosaminoglycans, specific and nonspecific proteases, lysosomal enzymes, biogenic amines, mitogens, growth factors, cytokines, chemokines, etc. (Galli et al. 2015; Mukai et al. 2018; Wernersson and Pejler 2014). A special characteristic of the biology of MCs is the high presence of proteases in comparison with other immunocompetent cells (Akula et al. 2020; Trivedi and Caughey 2010).

Depending on the expression of specific proteases, human MCs are classified into tryptase-positive, chymase-positive MCs, and MCs expressing both proteases (Pejler et al. 2007, 2010; Wernersson and Pejler 2014). Specific proteases comprise up to 25% of the secretome proteins and form the characteristic phenotype of MC depending on their organization and accumulation in the cytoplasm. From exerting biological effects on the structures of connective tissue (Atiakshin et al. 2020), tryptase and chymase are the most significant MC proteases, which are among the key factors influencing the phenotype formation of tissue microenvironment (Dell’Italia et al. 2018; Hernández-Hernández et al. 2012; Mulloy et al. 2017). This allows us to consider specific MC proteases as relevant research objects in morphological practice, not only as diagnostic markers, but also as a promising pharmacological target for therapy (Ammendola et al. 2016; Caughey 2016; Singh et al. 2016; Vitte 2015). The biological effects of tryptase and chymase depend on the secretion mechanism and have a selective effect on specific molecular microenvironment targets, modulating allergic and inflammatory reactions, angiogenesis, oncogenesis, causing remodeling of the extracellular matrix of connective tissue (Atiakshin et al. 2018a, b, 2020).

Due to the discovery of the hematopoietic cell carboxypeptidase gene, carboxypeptidase A3 has been shown to be an important marker for secretory granules, differentiation and MC phenotype (Goldstein et al. 1989; Pejler et al. 2009; Reynolds et al. 1992; Springman et al. 1995). At the same time, the detection of non-specific carboxypeptidases A1, A2, and B was carried out mainly to study pancreatic function, therefore the features of their distribution in MCs are still unexplored (Reznik and Fricker 2001; Tamura et al. 2018).

The cytomorphological examination of the bone marrow is an important diagnostic approach and an integral part of staging in mastocytosis. Cytomorphological aspects of MCs in smears of red bone marrow in systemic mastocytosis are very indicative of revealing a disease, have prognostic value and are important criteria for classifying a disease, not inferior to the effectiveness of molecular genetic studies, in particular, KITD816V mutation (Horny et al. 2014; Horny and Valent 2001; Pardanani 2019; Sperr et al. 2001; Valent et al. 2001, 2010).

Immunohistochemical studies of MCs significantly expanded the informational content of ongoing studies to identify their functional state (Horny et al. 2006, 2014; Valent et al. 2010, 2015). Immunohistochemical analysis of tryptase, chymase, and carboxypeptidase expression allows a better understanding of the development of mastocytosis and can be used both for the diagnosis of mastocytosis and as a promising pharmacological target. The aim of this study was a characterization of the protease profile of red bone marrow MCs in mastocytosis.

## Materials and methods

### Patients

8 mastocytosis patients were included in this study. Four patients were with indolent systemic mastocytosis, two patients with smouldering systemic mastocytosis, and two patients with aggressive systemic mastocytosis. Informed consent was obtained from all subjects. The samples were retrieved from the files of the Institute of Pathology, Ludwig-Maximilians University, Munich, Germany. These samples were redundant clinical specimens that had been de-identified and unlinked from patient information. Histological diagnoses were established according to the WHO classification (Arber et al. 2016; Horny et al. 2016; Valent et al. 2017). This study was conducted in accordance with the principles of the World Medical Association Declaration of Helsinki “Ethical Principles for Medical Research Involving Human Subjects” and approved by the Institutional Review Board of the Institute for Hematopathology, Hamburg, Germany.

### Tissue probe stainings

Trephine core bone marrow biopsy specimens were obtained from the posterior iliac crest. Routinely processed BM trephine biopsy specimens had been mildly decalcified in 0.07% (wt/vol) edetic acid (EDTA) for at least 8 h, and embedded in paraffin. Deparaffinised and rehydrated Sects. (2 µm thick) were subjected to antigen retrieval by heating in a steamer with sodium citrate buffer, pH 6.0, at 95 °C × 30 min. We reported earlier that endogenous Fc receptors in routinely fixed cells and tissue probes do not retain their ability to bind Fc fragments of antibodies (Buchwalow et al. 2011); therefore, blocking the endogenous Fc receptors prior to incubation with primary antibodies was omitted. After antigen retrieval, sections were immunoreacted with primary antibodies to tryptase, chymase, and carboxypeptidases (Table 1). Principally, immunohistochemical staining was performed according to the standard protocols described earlier (Buchwalow et al. 2005, 2018; Buchwalow and Boecker 2010).

**Table 1:** Primary antibodies used in this study

Antibodies	Host	Catalogue Nr	Dilution	Sourse
Tryptase	Mouse monoclonal Ab	#ab2378	1:3000	AbCam, United Kingdom
Tryptase	Rabbit monoclonal Ab	#ab151757	1:2000	AbCam, United Kingdom
Chymase	Mouse monoclonal Ab	#ab2377	1:2000	AbCam, United Kingdom
Carboxypeptidases A1, A2 and B	Rabbit monoclonal Ab	#ab 181,146	1:500	AbCam, United Kingdom

For brightfield microscopy, bound primary antibodies were detected with AmpliStain™ Horseradish Peroxidase (HRP) conjugates (SDT GmbH, Baesweiler, Germany) according to the manufacturers’ instructions (Boecker et al. 2013). The HRP label was visualized using a 3,3′-diaminobenzidine (DAB) substrate kit (Vector Laboratories, Burlingame, CA, USA). The nuclei were counterstained with hematoxylin.

For fluorescence detection, bound primary antibodies were visualized using secondary antibodies (purchased from AbCam, United Kingdom) conjugated with Cy3 or Alexa Fluor-488. Nuclei were counterstained with 4′,6-diamidino-2-phenylindole (DAPI, 5 µg/ml in PBS) for 15 s, and the sections were then mounted using VectaShield (Vector Laboratories, Burlingame, USA). The list of secondary antibodies and other reagents used in this study is presented in Table 2.

**Table 2:** Secondary antibodies and other reagents

Antibodies and other reagents	Source	Dilution	Label
Goat anti-mouse IgG Ab (#ab97035)	AbCam, United Kingdom	1/500	Cy3
Goat anti-rabbit IgG Ab (#ab150077)	AbCam, United Kingdom	1/500	Alexa Fluor 488
AmpliStain™ anti-Mouse 1-Step HRP (#AS-M1-HRP)	SDT GmbH, Baesweiler, Germany	Ready-to-use	HRP
AmpliStain™ anti-Rabbit 1-Step HRP (#AS-R1-HRP)	SDT GmbH, Baesweiler, Germany	Ready-to-use	HRP
4′,6-diamidino-2-phenylindole (DAPI, #D9542-5MG)	Sigma, Hamburg, Germany	5 µg/ml	w/o
VECTASHIELD^®^ Mounting Medium (#H-1000)	Vector Laboratories, Burlingame, CA, USA	Ready-to-use	w/o
DAB Peroxidase Substrat Kit (#SK-4100)	Vector Laboratories, Burlingame, CA, USA	Ready-to-use	DAB
Mayer’s hematoxylin (#MHS128)	Sigma-Aldrich	Ready-to-use	w/o

### Image acquisition

**Fig. 1:**
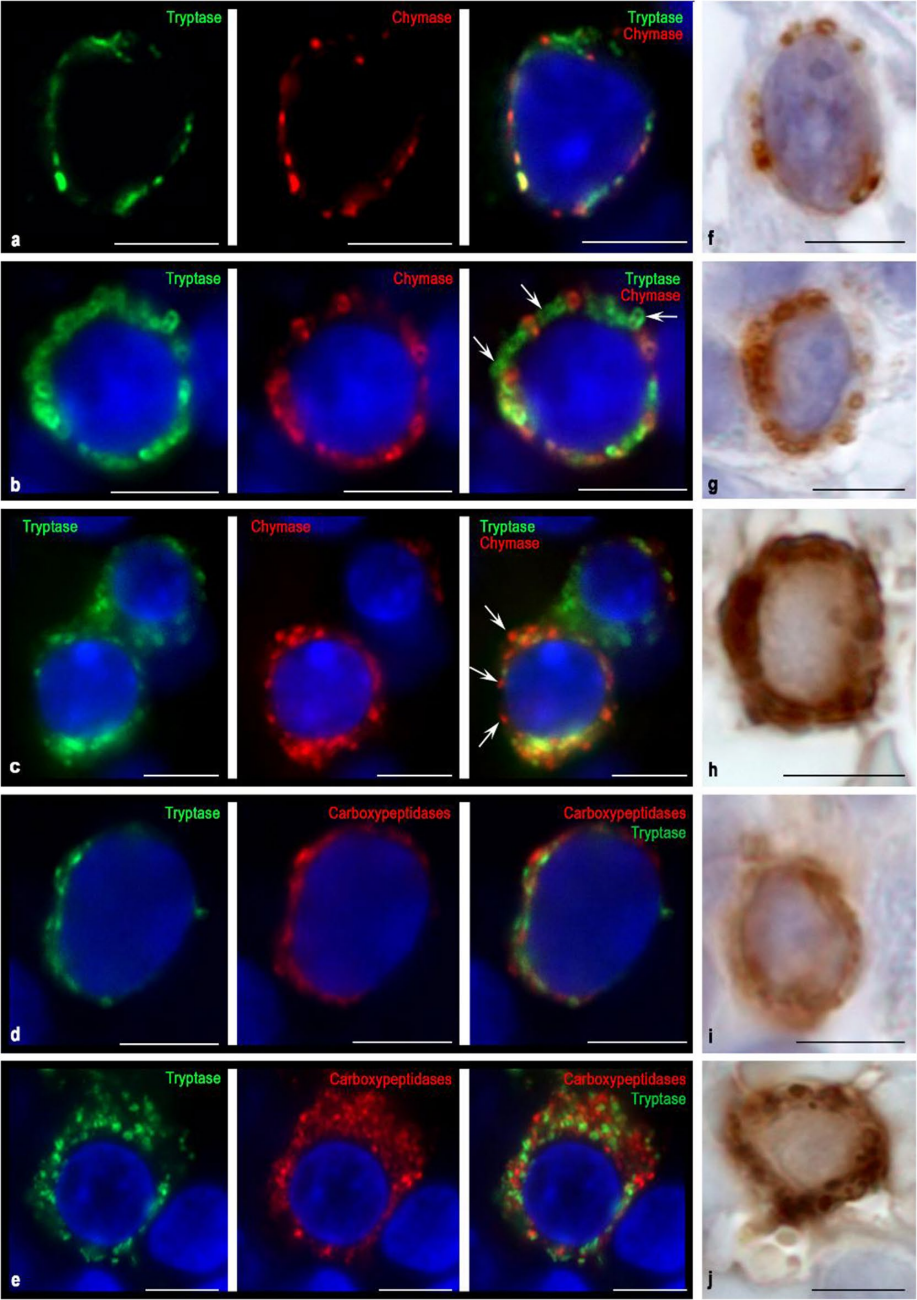
Cytological characterization of proteases in blast forms of MCs of red bone marrow during aggressive systemic mastocytosis. Tryptase was detected using immunolabeling with mouse monoclonal anti-tryptase AB (**d**, **e**, **h**, **i**) or rabbit monoclonal anti-tryptase AB (**a–c**). Chymase was detected using immunolabeling with mouse monoclonal anti-chymase AB (**a–c**, **f**, **g**). Carboxypeptidases was detected using immunolabeling with rabbit anti-carboxypeptidase A1 + A2 + B AB (**d**, **e**). Visualization of bound primary AB was performed using fluorochromes Alexa Fluor 488 and Cy3 (**a–e**) or DAB Chromogen (**f–j**). **a** Initial level of synthesis of specific proteases in MC. Typical secretory granules are absent. Chymase and tryptase are localized in a narrow zone of the perinuclear region. **b** Stage of specific proteases formation in granules of types II and III. Granules containing both tryptase and chymase are prevailing; granules containing the only tryptase are in a lower amount (marked with an arrow). **c** Accumulation of secretory granules in blast forms of MCs with the formation of MCs with different chymase contents. Granules containing exclusively chymase are marked with an arrow. **d** Mast cell in the initial stages of the formation of secretory material in the form of immature granules and granules of type I, in which tryptase is partially co-localized with carboxypeptidases. **e**—Blast form of a mast cell at the stage of differentiation into a more mature state. The cytoplasm is predominantly filled with carboxypeptidases localized extragranularly; tryptase takes up a smaller volume and is partially visualized in granules. **f–g** Single (**f**) and more numerous (**g**) chymase-positive granules in MC with clearly visible nucleoli. **h** Mast cell cytoplasm is filled with large tryptase-positive granules of type III. **i** Chromogenic detection of the initial stages of tryptase synthesis in the cytoplasm of the MC blast form. **j** Uneven accumulation of tryptase-containing granules in the cytoplasm of the MC blast form, while the perinuclear zone remains free of proteases. Bar 5 µm for the entire layout

**Fig. 2:**
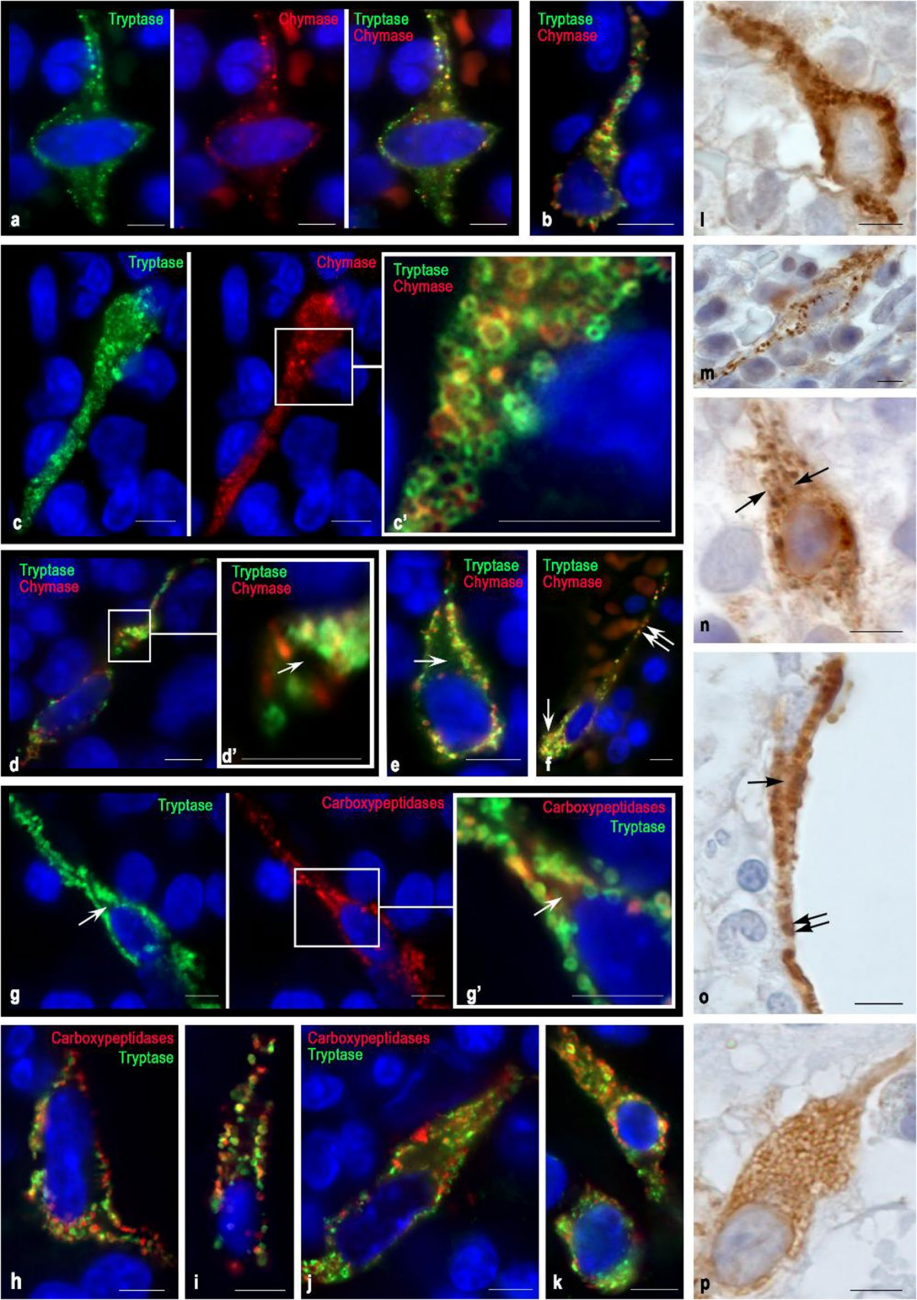
Specific proteases in MCs of type 1a of the red bone marrow in indolent systemic mastocytosis. Tryptase was detected using immunolabeling with mouse monoclonal anti-tryptase AB (**g–k**, **l–n**) or rabbit monoclonal anti-tryptase AB (**a–f**). Chymase was detected using immunolabeling with mouse monoclonal anti-chymase antibodies AB (**a–f**, **o–p**). Carboxypeptidases were detected using immunolabeling with rabbit anti-carboxypeptidase A1 + A2 + B AB (**g–k**). Visualization of bound primary AB was performed using fluorochromes Alexa Fluor 488 and Cy3 (**a–k**) or DAB Chromogen (**l–p**). **a**, **b** Various variants of hypogranulated MC with cytoplasmic outgrowths in which tryptase and chymase-positive secretory material are visualized. **c**—Elongated MC with an eccentric position of the nucleus and well-defined secretory granules of type II and III; the peripheral annular part of granules is tryptase and/or chymase positive. **d** Spindle-shaped MC with the formation of cytoplasmic outgrowths at considerable distances from the nucleated region of the cell. **d’** Cross-section of the cytoplasmic outgrowth. Granule-containing peripheral region and protease-free central part are (marked with an arrow). **e** Hypogranulated MC with a cytoplasmic outgrowth in which proteases are localized in the peripheral region, whereas the central region is free of proteases (indicated by an arrow). **f** Hypogranular mast cell with the formation of two morphologically different poles. The wide pole (indicated by the arrow) is filled with granules with the equal expression of tryptase and chymase. A narrow cytoplasmic outgrowth (indicated by a double arrow) contains fewer specific proteases and accompanies a vessel of the microvasculature at a considerable distance. **g** Elongated mast cell with a cytoplasmic outgrowth of a considerable length. The peripheral region of the appendix contains proteases, while the central region is free of granular secretory material (indicated by the arrow). **h**, **i**, **j** Various forms of localization of tryptase and carboxypeptidases in MC with elongated nuclei, cytoplasmic outgrowths, and the peripheral arrangement of secretory granules in them. Both tryptase- or carboxypeptidase-positive granules are visible, as well as granules with simultaneous expression of both proteases. **k** MC with different ratios of tryptase and carboxypeptidases expression. **l** Tryptase-positive MC with a cytoplasmic outgrowth containing tryptase-positive granules. **m** Elongated MC with a small content of secretory granules both in the perinuclear region and in the cytoplasmic outgrowths. **n** Hypogranular MC. In the cytoplasm, single secretory granules and longitudinally directed small structural formations that are immunopositive to tryptase are obvious (indicated by an arrow). **o** Spindle-shaped MC neighbouring an adipocyte, filled with a large number of chymase-positive granules. In the perinuclear cytoplasm, the central region is free of granules (indicated by an arrow), while the distance from the nucleus, the size of granules is comparable with the volume of the cytoplasm (indicated by a double arrow). **p** Hypogranulated chymase-positive MC with the formation of a narrow cytoplasmic outgrowth on the periphery. Bar 5 µm for the entire layout

**Fig. 3:**
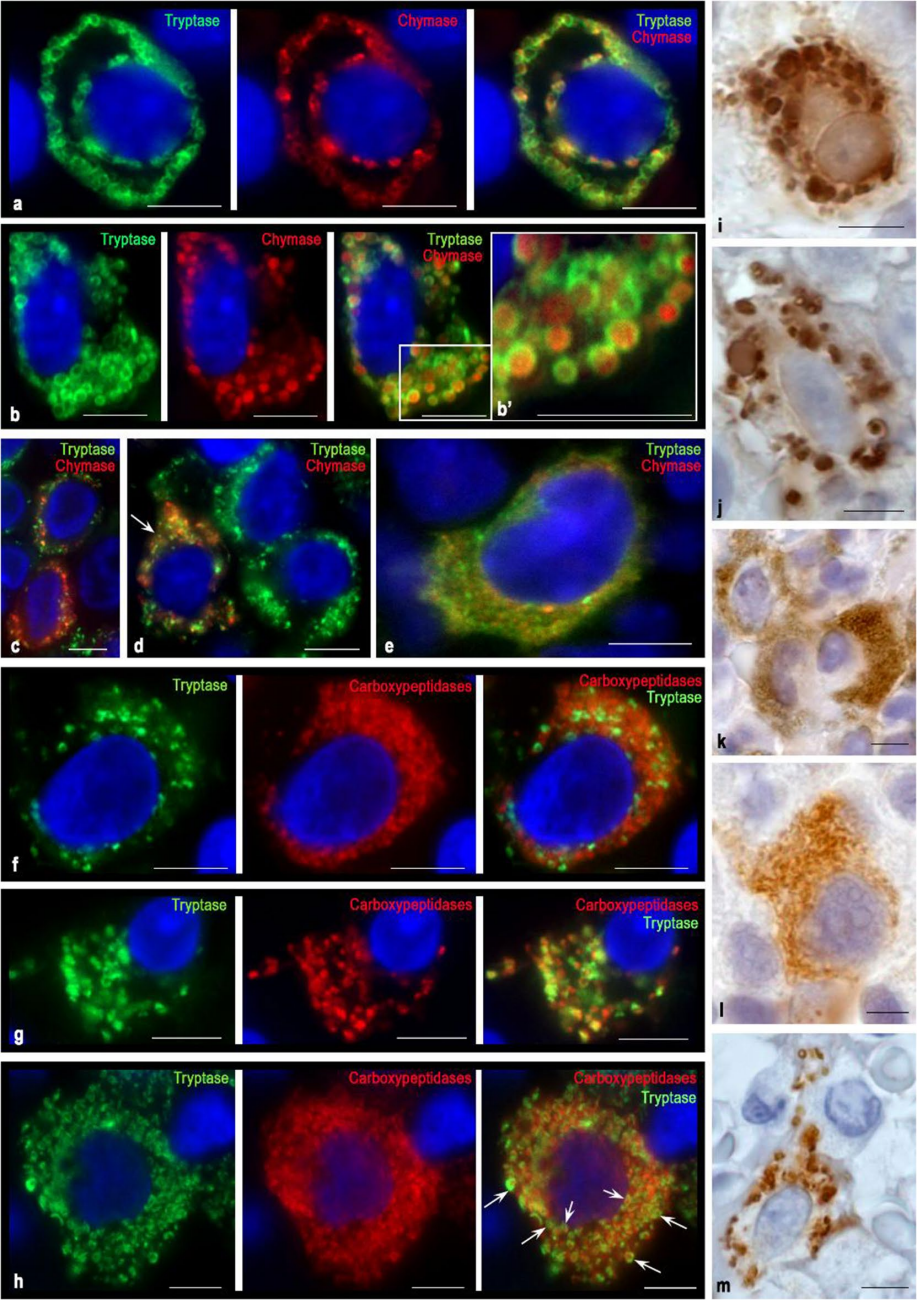
Cytological features of the distribution of proteases in MCs of type 1b in the red bone marrow in smouldering systemic mastocytosis. Tryptase was detected using immunolabeling with mouse monoclonal anti-tryptase AB (**a–e**, **i–k**) or rabbit monoclonal anti-tryptase AB (**f–h**). Chymase was detected using immunolabeling with mouse monoclonal anti-chymase AB (**a–e**, **l–m**). Carboxypeptidases were detected using immunolabeling with rabbit anti-carboxypeptidase A1 + A2 + B AB (**f–h**). Bound primary AB was visualized using fluorochromes Alexa Fluor 488 and Cy3 (**a–h**) or DAB Chromogen (**i–m**). **a** Localization of protease granules in the peri-plasmalemmal and perinuclear regions of the MC. Tryptase and chymase are located on the periphery of granules of type III. **b** A large number of mature secretory granules in the MC cytoplasm with uneven accumulation. High accumulation of chymase in the MC secretory granules with filling the central region. **c** Variable content of chymase in hypogranular MCs. **d** MC with high chymase expression (indicated by an arrow) neigbouring two tryptase-positive MCs. **e** Hypogranulated MC with an eccentrically positioned nucleus, with the simultaneous content of specific proteases in the cytoplasm. **f** Hypogranulated MC with predominant localization of carboxypeptidases outside of tryptase-positive granules and an eccentric arrangement of the nucleus. **g** Localization of tryptase and carboxypeptidases in MC granules with an eccentric position of the nucleus. **h** Tryptase-positive MC with a high content of carboxypeptidases, co-localized intragranularly with tryptase (indicated by an arrow) or extragranularly in the cytoplasm. Neighbouring to another tryptase-positive MC is observed. **i** Localization of tryptase in various granules, which have a pronounced polymorphism. Near the nucleus with a clearly visible nucleolus, a cytoplasm region, not filled with granules (presumably occupied by the Golgi complex) is identified. **j** Pronounced polymorphism of tryptase-positive granules in MC with an elongated nucleus. **k** A group of tryptase-positive hypogranular MC with a low level of protease in the perinuclear region. **l** Absence of mature type III protease granules in a hypogranulated chymase-positive MC with an eccentrically located nucleus. **m** Elongated chymase-positive MC whose cytoplasm is unevenly filled with mature type III secretory granules. Bar 5 µm for the entire layout

**Fig. 4:**
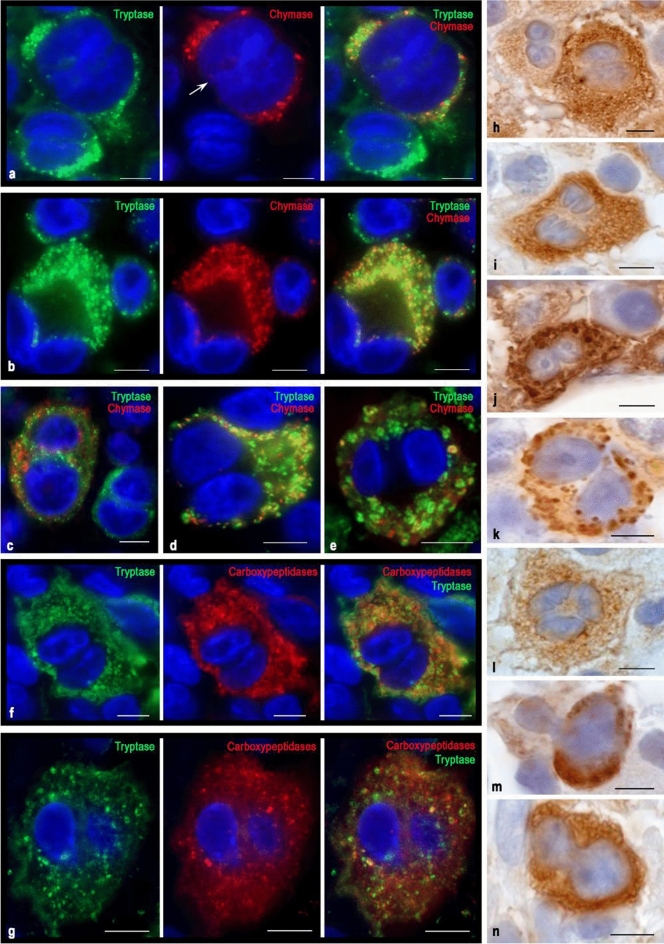
Cytotopography of proteases in MCs of type 2 of the red bone marrow in aggressive systemic mastocytosis. Tryptase was detected using immunolabeling with mouse monoclonal anti-tryptase AB (**f–g**, **h–j**, **n**) or rabbit monoclonal anti-tryptase AB (**a–c**). Chymase was detected using immunolabeling with mouse monoclonal anti-chymase AB (**a–c**, **k–m**). Carboxypeptidases were detected using immunolabeling with rabbit anti-carboxypeptidase A1 + A2 + B AB (**f–g**). Bound primary ABs were visualized using fluorochrome Alexa Fluor 488 and Cy3 (**a–g**) or DAB Chromogen (**h–n**). **a**, **b**, **c** – Variants of the localization of hypogranular mast cells with unequal expression of tryptase and chymase. **a** Large MC containing tryptase and chymase with a segmented nucleus (indicated by an arrow) in a contact with a binuclear tryptase-positive mast cell that does not express chymase. **b** Large binucleated MC with simultaneous expression of tryptase and chymase contacting with blast MCs, one of which contains only tryptase (top), and the other both proteases (right). **c** A large hypogranulated binucleated MC containing both specific proteases is localized near a smaller binucleated MC cell that does not express chymase. **d**, **e** Variants of co-expression of tryptase and chymase in hypogranulated (**d**) and granulated (**e**) binuclear MC. **f**, **g** MCs with a high (**f**) and low (**g**) expression of tryptase and carboxypeptidase. **h** Contact of hypogranulated binuclear MCs with high (right) and moderate (left) tryptase expression. The pronounced nucleoli in the nuclei of an MC with lower protease content are noteworthy. **i** Hypogranulated tryptase-positive TK with abnormal shape of the nuclei. **j** A large MC with large nucleoli in the nuclei and with well-defined mature tryptase-positive granules of type III. **k** Binuclear MC with the expression of chymase, formed into large secretory granules. A large nucleolus is visible in one of the nuclei. **l** Hypogranulated chymase-positive MC with well-formed nucleoli in the nuclei. **m** Abnormal form of the nucleus in a chymase-positive MC with a small content of large secretory granules. **n** Binuclear hypogranulated tryptase-positive MC. Bar 5 µm for the entire layout

**Fig. 5:**
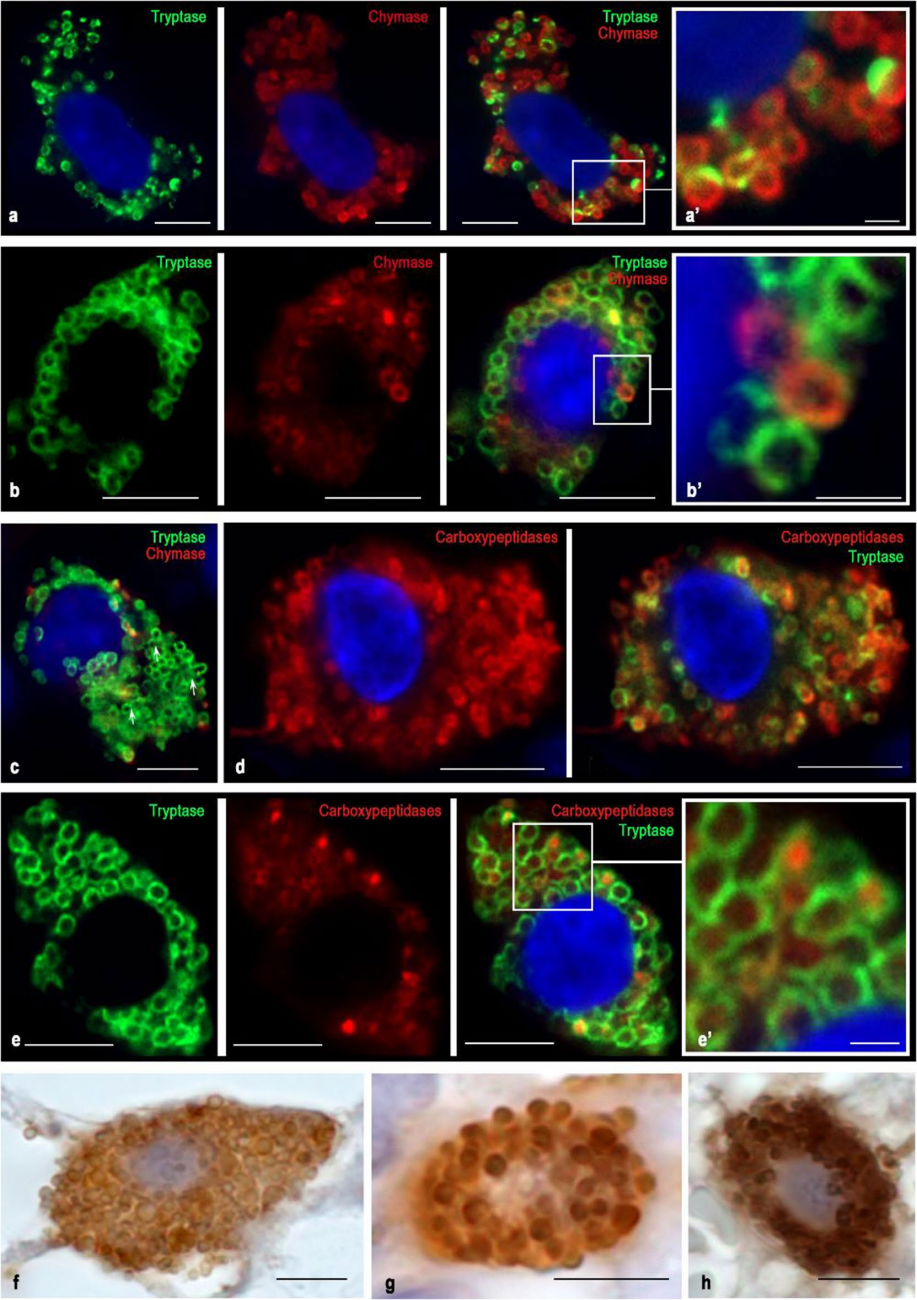
Proteases in mature mast cells of red bone marrow in patients with indolent systemic mastocytosis. Tryptase was detected using immunolabeling with mouse monoclonal anti-tryptase AB (**d–e**, **h**) or rabbit monoclonal anti-tryptase antibodies AB (**a–c**). Chymase was detected using immunolabeling with mouse monoclonal anti-chymase antibodies AB (**a–c**, **f–g**). Carboxypeptidases were detected using immunolabeling with rabbit anti-carboxypeptidase A1 + A2 + B AB (**d–e**). Visualization of bound primary AB was performed using fluorochromes Alexa Fluor 488 and Cy3 (**a–e**) or DAB Chromogen (**f–h**). **a** A mast cell with a high level of chymase expression. Chymase prevails both in the number of granules in the MC cytoplasm and the intragranular content. In granules, chymase is concentrated on the periphery (**a’**). **b** A MC with approximately equal contents of tryptase and chymase. Granules containing exclusively chymase or tryptase are detected, as well as with simultaneous protease content (**b’**). Proteases are localized on the periphery of mature granules of type III. **c** A MC with a predominance of tryptase-positive granules, some of which tend to fusion with each other (marked by an arrow). **d** A tryptase-positive MC with an intra- and extra-granular carboxypeptidase content. Tryptase is localized exclusively on the periphery of the granules. **e** A tryptase-positive mast cell with predominant intragranular localization of carboxypeptidases in the central region of the granules (**e’**). **f**, **g**—The mast cell cytoplasm is filled with large chymase-positive granules, some of which are secreted by the exocytosis mechanism. One can see single freely lying secretory granules in the extracellular matrix (**f**). **h** A tryptase-positive mast cell with low secretory activity. Bar 5 µm for the entire layout and 1 µm for the **a’**, **b’**, **e’**

**Fig. 6:**
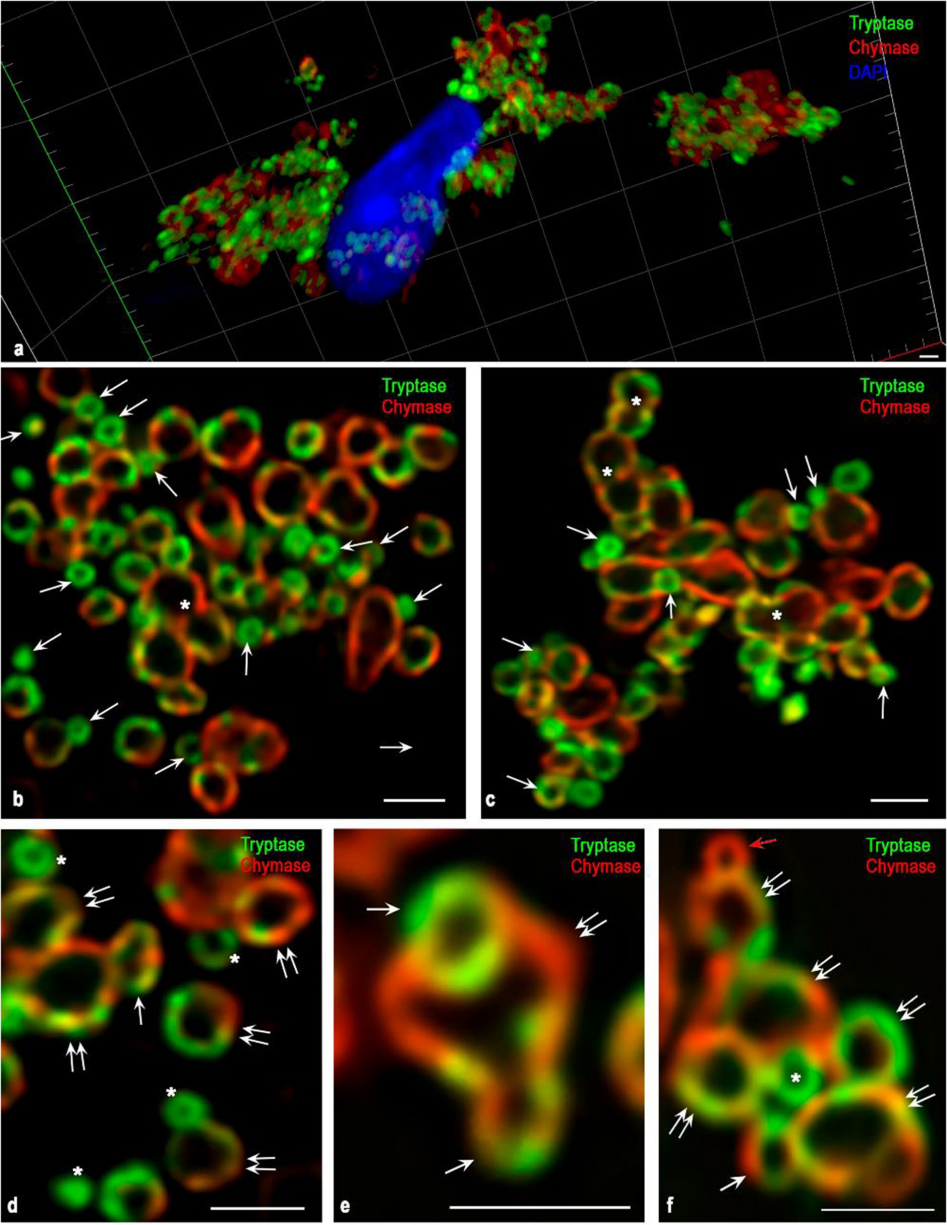
Cytotopography of specific proteases in the secretion of an atypical mast cell of type 1a with indolent systemic mastocytosis. Tryptase was detected using immunolabeling with rabbit monoclonal anti-tryptase AB. Chymase was detected using immunolabeling with mouse monoclonal anti-chymase AB. Visualization was performed using fluorochrome Alexa Fluor 488 and Cy3. Images were obtained using a confocal scanning microscope ZEISS LSM 880/Airyscan equipped with a Zeiss Plan-apochromat objective 63x/1.40 oil. **a** A general plan for the localization of tryptase and chymase in the secretion of MC. **b, c** Various options for the localization of chymase and tryptase in secretory granules. The cytoplasm contains a large number of small granules of type II (indicated by an arrow) contacting with each other and adjacent to large mature secretory granules of type III. In secretory granules, proteases are localized on the periphery. Well-pronounced wide variability of intragranular co-localization of tryptase and chymase. Some mature granules have visually distinguishable anastomoses with each other (indicated by an asterisk). **d–f** Morphological equivalents of different variants of fusion of immature secretory granules of type II with large mature granules (indicated by a double arrow) during post-translational modification of specific proteases. At the stage of an immature granule, granules can contain mainly chymase (red arrow), tryptase (asterisk), or both specific proteases (white arrow). Bar 1 µm for the entire layout

Immunostained tissue sections were observed on a ZEISS Axio Imager.A2 equipped with a Zeiss alpha Plan-Apochromat objective 100x/1.46 Oil DIC (UV) VIS-IR and AxioCam digital microscope cameras (Axiocam 506 color and Axiocam 503 monochrome CCD). Captured images were processed with the software program ZEN 2.3 (Carl Zeiss Vision, Germany) and submitted with the final revision of the manuscript at 300 DPI (Figs. 1, 2, 3, 4, 5). Two- and three-dimensional images with a higher optical resolution were obtained using a confocal scanning microscope ZEISS LSM 880/Airyscan equipped with a Zeiss Plan-apochromat objective 63x/1.40 oil (Fig. 6).

### Controls

Control incubations were: omission of primary antibodies or substitution of primary antibodies by the same IgG species (Dianova, Hamburg, Germany) at the same final concentration as the primary antibodies. The exclusion of either the primary or the secondary antibody from the immunohistochemical reaction, the substitution of primary antibodies with the corresponding IgG at the same final concentration resulted in a lack of immunostaining.

## Results

We assessed protease expression in various types of MCs: metachromatically granulated blast, atypical MC type 2, atypical MC type 1, and typical (mature) tissue MC (Sperr et al. 2001; Valent et al. 2001). Type 1 was differentiated into more mature MC (1a type) and 1b type (more immature cells) (Sperr et al. 2001). In this work, we used the classification of secretory granules, on the basis of which, according to ultrastructural characteristics, three types are distinguished according to the features of the structural composition, size, and degree of maturity (Blank 2011; Blank et al. 2014; Pejler et al. 2007; Wernersson and Pejler 2014).

Unable to identify the least mature type I secretory granules resembling endosomes or lysosomes with luminal vesicles, we discuss mainly types II and III secretory granules. Formed in the course of homotypic and heterotypic fusions, granules of types II and III reflect sequential stages of selective accumulation of secretome components together with an increase in size to 1 μm or more (Azouz et al. 2014; Blank et al. 2014; Raposo et al. 1997). The stages of granule biogenesis are discussed in more detail in the Discussion section.

### Metachromatically granulated blast-like cells

Blast forms of MCs were small rounded cells of 5–7 μm in size with a predominant nucleus volume. The cytoplasm contained tryptase-positive and chymase-positive granules of type II and III and constituted a narrow rim around the nucleus (Fig. 1). At the earliest stages of blast-like MC differentiation, protease-positive progranular formations or type I secretory granules could be seen in close contact with the nuclear membrane (Fig. 1a, d, i). At further stages of maturation, single protease-containing granules of type II or III were formed (Fig. 1f, g, i, j).

Multiple immunolabeling showed the existing identity of the synthesis of tryptase and chymase in blast forms of MC (Fig. 1b, c). In this case, the presence of specific proteases can be detected at the stage of type II secretory granules. From protease specificity, tryptase, and chymase co-localization was most often detected in blast-like MC granules. The formation of type III secretory granules is accompanied by a special folding of specific proteases along the periphery of the granule contents (Fig. 1b). As they mature, the blast forms of MC reveal individual characteristics of the protease phenotype, which can be expressed both in the spatial-quantitative ratio of tryptase and chymase in a single granule and the total number of secretory granules. Thus, in the early stages of development, the formation of MC is accompanied not only by phenotypic characteristics but also by the characteristic folding of specific proteases inside secretory granules. The specific tissue microenvironment of the red bone marrow is an important factor for the expression of MC proteases and determines the ratio of blast-like MC with a different protease phenotype (Fig. 1c).

Interesting results were obtained by double immunolabeling of tryptase and carboxypeptidases. It turned out that the expression of carboxypeptidases was found in all blast forms, and most often they occupied a larger area in the cytoplasm compared to specific proteases (Fig. 1d). During the formation of the secretory granules of type II and subsequently type III, not always was intragranular co-localization of tryptase and carboxypeptidases observed. Figure 1e clearly shows that during the differentiation of a blast-like mast cell, the bulk of carboxypeptidases was located outside tryptase-positive granules. These facts indicates that at the stage of MC blast forms, the main stages of specific proteases processing are carried out extragranularly and reflect the MC activity level on post-translational proteins modification for various purposes. Less mature type II secretory granules do not have the level of intragranular tryptase and chymase processing characteristic of type III mature granules. Thus, the expression of specific proteases at the initial MC differentiation stages is quite variable and closely related to the specific tissue microenvironment state of the red bone marrow.

### Atypical mast cell type 1a

Type 1a mast cells are the morphological equivalent of the most differentiated atypical MC variant of the red bone marrow in mastocytosis. They acquire an elongated shape and demonstrate pronounced cytoplasmic outgrowths (extensions), which can extend over considerable distances (Fig. 2). In the perinuclear zone of the cytoplasm of such MCs, proteases can appear in mature type III secretory granules (Fig. 2c, g, o).

**Fig. 7:**
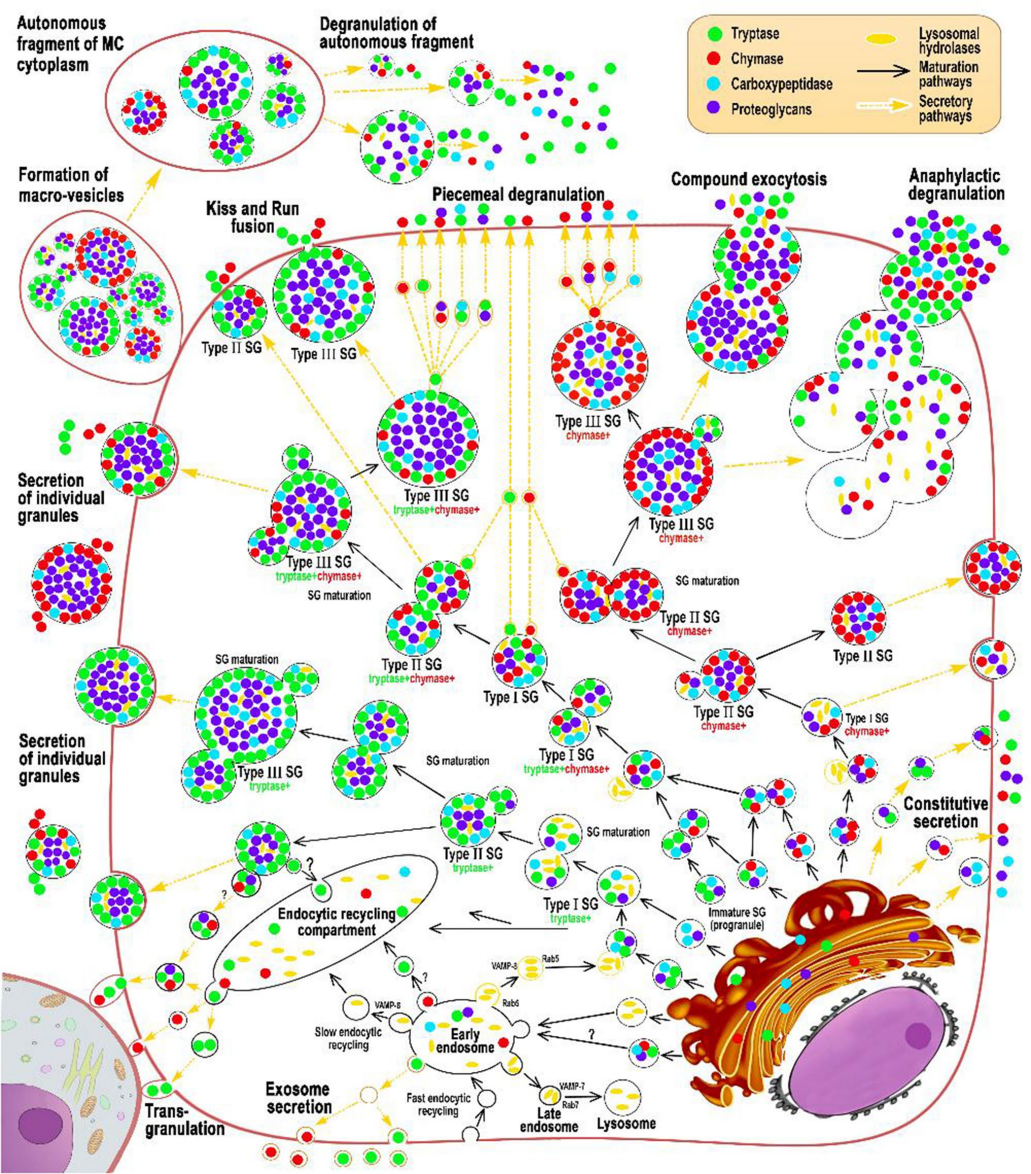
Cytotopography and secretory pathways of mast cell proteases in red bone marrow in systemic mastocytosis. The scheme (Fig. 7) shows the main stages of post-translational modification of proteases, taking into account their cytotopography, intragranular localization, and secretory mechanisms. Protease biosynthesis starts in the granular endoplasmic reticulum of mast cells (MCs) and continues in the Golgi apparatus (GA), where immature granules are formed. According to morphometric characteristics and the specificity of the intragranular folding of proteases, secretory granules can be divided into 3 types. Secretory granules of type I are formed after the fusion of the lysosome with the granules coming from the Golgi complex having the smallest size and the lowest protease content. As granules are enlarged through homotypic fusion, granules of type II are formed with a size of 0.2–0.4 μm; a characteristic feature is the laying of proteases on the periphery of the secretory granule, while proteoglycans in the central region. Secretory granules of type II are chymase + , tryptase + , as well as with the simultaneous content of specific proteases. As a result of the completion of maturation stages, type III secretory granules with sizes of 0.5 μm or more are formed, which are characterized by the largest volume of secretome while maintaining the peripheral localization of tryptase and chymase in the form of a ring. These MC granules contain also carboxypeptidases; their content and cytotopography are bound with tryptase and chymase biogenesis. Hypogranulated atypical MCs red bone marrow formed during mastocytosis is characterized by the prevalence of type I and II secretory granules in the cytoplasm that are morphologically indistinguishable and lead to diffuse immunohistochemical staining of the cytoplasm. The main mechanisms for the removal of specific proteases from hypogranulated MCs into the extracellular matrix are pacemaker secretion, transgranulation, and exosome formation. In the secretory granules of type II or III in atypical MCs, protease secretion is possible using the “Kiss and run” mechanism, exocytosis of individual granules, or lacing of macrovesicles, which for a long time retain autonomous secretory activity in a specific tissue microenvironment of the red bone marrow

When the proteases intracellular organization is completed at the secretory granules level of type I or II, a hypogranulated MC phenotype is formed (Figs. 2a, e, j, n, p, 6, 7). Mature or immature secretory granules with specific proteases can be seen distally from the nucleus along the outgrowths of the cytoplasm while occupying the peripheral position, actually adjacent to the plasmalemma (Fig. 2a, b, d, e, c, h, i). Figure 2d demonstrates a transverse section of the cytoplasmic outgrowth characterizing the cytotopography of specific proteases in atypical type 1a MCs. Here, the central part free of secretory material is visible. The secretome may be tryptase-positive or contain both specific MC proteases (Fig. 2d).

In atypical type 1a MCs, granules are formed in the perinuclear region, and then transported over fairly considerable distances through the cytoplasm via the corresponding intracellular transport systems. Figure 2n can serve as the morphological evidence of this process, where the directed transport of tryptase into the peripheral region of the cytoplasmic outgrowth of MC is well visualized. Proteases contained in granules of various maturity stages and progranules formations can be transported along the periplasmalemma region (Fig. 2g, h, i, o). Apparently, the granules occupy certain loci throughout the cytoplasmic outgrowth and secrete proteases by the “kiss and run” mechanism. Interestingly, in some cases, the cytoplasmic outgrowths may have a considerable length (Fig. 2f). This might be a manifestation of the mechanism determining the MC influence on the endothelium functional activity, as well as on the entry of proteases into the bloodstream. The carboxypeptidases presence in the cytoplasm of atypical type 1a MCs outgrowths indicates the universal mechanism of these proteases work, regardless of their disposition in the cell and the secretome proteins processing at a considerable distance from the nucleus. Localization of carboxypeptidases directly in the granules may indicate a partial cessation of post-translational modification of proteins in the cytoplasm after their entrance into granules. Notably, the high content of carboxypeptidases in the cytoplasm indicates an active level of these enzymes expression in MC. However, in the case of hypogranulated MC, the high carboxypeptidases content may indicate an active modification of enzymes with the acquisition of biological activity and rapid excretion from the cell. Obviously, a decrease in the carboxypeptidases representation is evident of the secretome components expression level reduction, including specific proteases, and reflects the formation of a state of relative calm in biosynthetic processes.

It should be noted that the different cytotopographic localization of tryptase and carboxypeptidases in hypogranular forms of MC is a frequent morphological finding. Significant differences exist in the proteases content in the MC of hypogranulated and granular forms. Most likely, the hypogranular forms are a more unfavorable variant of the course of mastocytosis due to the rather high intensity of protease released into the extracellular matrix using piecemeal secretion. In this case, proteases are not formed into classical mature granules and are not stored in the cytoplasm of MC, but undergo rapid secretion. This fact is considered an unfavorable diagnostic sign of mastocytosis associated with the multifaceted biological effects of tryptase and chymase.

At the same time, by indolent forms of mastocytosis, atypical type 1a MCs of the spindle-shaped form with outgrowths of the cytoplasm contain mature type III secretory granules (Fig. 2c, g, l). This is an obvious morphological sign of a more favorable course of the disease and a slower proteases release into the extracellular matrix is evident with the wide palette provision of biological and undesirable specific proteases pathophysiological effects.

### Atypical mast cell type 1b

This type of cell is less differentiated compared to type 1a MC. Two types of such cells can be distinguished: granular and hypogranular. In the first case, such MCs may resemble the structure of mature forms of MCs. However, the elongated shape of the nucleus and its eccentric position, as well as a possible pronounced polymorphism of the granules, indicate an atypical MC phenotype (Fig. 3). Protease-containing granules can be of various sizes, even if they are not numerous in the cytoplasm (Fig. 3i, g, m). Some granules have abnormally large sizes, which indicate a disturbance of the secretion mechanism and a distortion of the release of specific proteases into the extracellular matrix (Fig. 3i, j). At the same time, the presence of ordinary granules in such cells compensates for the MC regulatory potential failure by specific proteases. Depending on the course of mastocytosis, the MC granular and hypogranulated forms ratio changes with an increase in the MC and may indicate an unfavorable course of mastocytosis.

Unlike type 1a, type 1b MCs do not form significant outgrowths of the cytoplasm, but they can form wide protrusions (Fig. 3b, l). At the same time, the perinuclear cytoplasmic region presence in many hypogranulated cells which is almost immunonegative for specific TK proteases, suggests very active processes of post-translational specific proteases modification in the Golgi complex (Fig. 3b, d, k). Such cells can represent the majority of MC in the red bone marrow population with aggressive forms of mastocytosis.

At the same time, it is possible to suggest the secretion of proteases in granular atypical type 1b MCs using the “kiss and run” mechanism, in which mature granules line up along the plasma membrane, come into contact with it and form selective transport of the necessary secretory components (Figs. 3a, 7). Granules of atypical type 1b MCs most often contain both tryptase and chymase. In the non-aggressive course of mastocytosis, extensive protease-negative zones are formed in the cytoplasm of type 1b MC, which may indicate low activity of protease secretion into the extracellular matrix.

In hypogranulated atypical type 1b MCs, proteases are diffusely distributed over the cytoplasm, which implies active piecemeal degranulation of proteases into the extracellular matrix. Unfortunately, immunohistochemical confirmation of microvesicular transport is very difficult due to the small size of secretory formations released from the cell (Fig. 3e). However, it should be noted that the hypogranulated cells are quite clearly differentiated into tryptase-positive, and MC, which express both tryptase and chymase (Fig. 3c, d, e). In some cases, individual mature granules are observed in hypogranulated cells, which may contain both tryptase and chymase.

Noteworthy, the protease profile of atypical type 1b MCs is characterized by a high content of carboxypeptidases B (Fig. 3f, h). This indicates a high level of post-translational protein modification processes for constitutive exocytosis or induced secretion. Three variants of cytotopography of carboxypeptidases can be noted: mainly outside the granules, in granules, and combined localization. Most likely, this is associated with the stage of the intracellular process in the biogenesis of specific proteases. The presence of small progranular formations that do not contain carboxypeptidases with their high concentration in the cytoplasm indicates the actively proceeding extragranular stage of the processing of specific proteases (Fig. 3f). Localization of carboxypeptidases exclusively in mature MC granules may indicate proteases modification suspension in the cytoplasm and the intragranular proteases local modification (Fig. 3g). Finally, the carboxypeptidases detection both in the cytoplasm and inside mature granules indicates an actively ongoing process of granule biogenesis at all stages of proteases processing (Fig. 3h).

### Atypical mast cell type 2

Atypical type 2 MCs are a morphological sign of the progression of mastocytosis. Their striking feature is the segmentation of the nucleus (nuclei bi- or polylobed) (Fig. 4). Interestingly, a rather atypical shape of the nuclei or their segments, which forms the “dentation” of the contours, was observed (Fig. 4i). In some cases, protease-positive mast cells had atypical segmentation in the form of disproportionately small outgrowths or had nuclear segments of different sizes (Fig. 4l, m, n). The sizes of such MCs can differ significantly from each other in the range from 10 to 20 µm. Also, the nuclear-cytoplasmic ratio can be different, from high to low. Having in some cases a small volume of cytoplasm, atypical type 2 MCs contain very small secretory granules (Fig. 4a). Small granules are evenly distributed in the cytoplasm, without the formation of local clusters. The cytoplasm of MCs can be immunopositive for tryptase and chymase not only within the granular formations or granules but also between them. Often different in size, type 2 MCs are closely adjacent to each other (Fig. 4a, b). Moreover, neighboring MCs may differ in the expression of specific proteases from each other. For example, type 2 MC with simultaneous expression of tryptase and chymase can be contacted with a smaller tryptase-positive MC (Fig. 4a, c). Also, large MCs of type 2 may also affect other types of MCs, obviously affecting further differentiation, protease expression, and secretory activity (Fig. 4b).

The intracytoplasmic distribution of proteases can be almost uniform. However, immature granules of type 2 may be of three variants: tryptase-positive, chymase-positive, and also with the simultaneous content of both proteases (Fig. 4d). Binuclear MCs are sometimes granular and have mature secretory granules with characteristic edge localization of tryptase and chymase in the form of a ring, however, their number may depend on the specificity of the disease (Fig. 4e, j, k). Large mature granules can be represented in various numbers, from a single (Fig. 4k) to the almost complete filling of the cytoplasm (Fig. 4j). Despite the generally high expression of specific proteases in hypogranulated type 2 MC, it can vary from moderate to high (Fig. 4h).

Binuclear cells were typically characterized by high levels of carboxypeptidases (Fig. 4f, g).

### Typical tissue MCs

Typical mature MCs of the red bone marrow were well-granulated, large cells, reaching a size of 15–20 µm. Their nuclei, as a rule, had a rounded shape and were located in the center of the cell. Specific proteases were formed into well-distinguishable granules about 1 μm in size; their number was significantly high and they could fill almost the entire cytoplasm (Fig. 5).

In mature cells, the predominant intragranular localization of specific proteases became quite obvious. As a rule, both tryptase and chymase were located on the periphery of the granule, which was earlier shown by us in MCs of other organs (Fig. 5a’, b’) (Atiakshin et al. 2018a, 2019). At the same time, mature MCs separate phenotypes existence of three variants in the red bone marrow was noted: with the chymase predominance, the prevalence of tryptase, or the specific proteases simultaneous maintenance in relatively equal amounts (Fig. 5a, b, c). The mode of the intragranular distribution of proteases is noteworthy. In particular, epifluorescence microscopy gives the impression of a larger area occupied by the chymase compared with tryptase-positive material (Fig. 5a, b). Carboxypeptidases were found both inside the granules and outside the granules in the cytoplasm of typical tissue MCs (Fig. 5e, d).

Sometimes protease-positive granules were visualized autonomously in a specific tissue microenvironment of the red bone marrow. This indicates the possibility of their autonomous existence in the extracellular matrix sometime after secretion, which allows the delayed regulatory functions of tryptase and chymase to be realized.

## Discussion

This study showed that each type of atypical MC in mastocytosis has its protease profile, which is characterized by the ratio of proteases content within a cell. The total set of MCs determines the integral functional potential of tryptase and chymase proteases in the bone marrow. Three MC states can be distinguished, depending on the cytotopographic features of the distribution of carboxypeptidases. We have also revealed the expression of nonspecific carboxypeptidases A1, A2, and B in all MCs. It should be noted that the Anti-Carboxypeptidase A antibody (#ab173283) raised against pancreatic carboxypeptidase A was completely ineffective for detecting MCs in the red bone marrow. At the same time, the use of Anti-Carboxypeptidase A1 + A2 + B antibody (#ab181146) resulted in a convincing staining of MCs. According to the manufacturer, this antibody should be specific for carboxypeptidase B, in addition to carboxypeptidases A1 and A2.

Of the known carboxypeptidases of the pancreas A1, A2, and B, it is the latter that has a higher similarity to the carboxypeptidase of A3 of MCs (Enoksson et al. 2011; Goldstein et al. 1989; Pejler et al. 2009). The amino-terminal amino acid sequence of MC carboxypeptidase demonstrated 65% positional identity with human pancreatic carboxypeptidase B (Goldstein et al. 1989). It was shown that carboxypeptidase A3 of MCs is more similar to carboxypeptidase B in terms of protein structure and molecular genetic features (Goldstein et al. 1989; Reynolds et al. 1992). Thus, despite the use of an antibody for selective detection of carboxypeptidase B in the work, it is most likely that carboxypeptidase A3 was detected in the MCs of the red bone marrow of patients with mastocytosis, which should be taken into account when interpreting the results obtained in the present work.

### Cytotopography of processing and secretion of specific proteases in mastocytosis: formation of a hypogranulated MC phenotype

The pathogenesis of mastocytosis is closely associated with the biogenesis of specific proteases. About 5% of the genome encodes information on human proteases, and the MC protease mRNA content is comparable to the content of housekeeping genes transcripts. This reflects the high content of proteases in the cytoplasm of MCs, which can present more than 25% of the total MC protein (Akula et al. 2020; Caughey 2007, 2011, 2016; Pejler et al. 2010; Schwartz 1990; Schwartz et al. 1987a, b; Shea-Donohue et al. 2010). Biogenesis of specific proteases in mastocytosis can be manifested by the formation of a hypogranulated MC phenotype. It should be noted that the initial stages of the processing of tryptase and chymase during mastocytosis are obviously carried out along the canonical path. The classical model of the formation of MC secretory granules suggests that after protease synthesis in rough endoplasmic reticulum they enter the Golgi complex, where they undergo post-translational modification and are packaged in small-sized granular formations surrounded by a membrane (Hammel and Meilijson 2015; Lorentz et al. 2012). These granules containing specific and non-specific proteases complex with proteoglycans in the cytoplasm can merge, and after combining with acid hydrolases from type early endosomes, type I secretory granules are formed (Fig. 7). Morphologically, they are indistinguishable, small in size, and are further combined with the formation of secretory granules of type II, reaching visualized sizes from 0.2 to 0.4 μm (Fig. 7). At this stage of maturation, type II secretory granules have a certain secretome composition, including glycosaminoglycans, proteases, lysosomal enzymes, etc. Type II secretory granules are enlarged in size by combining with similar or secretory type I granules (Figs. 6, 7). Their further maturation leads to a further increase in volume and is accompanied by the formation of type III secretory granules with sizes of 0.4–1 µm with the accumulation of a unique composition of specialized secretome components. Such granules can be tryptase-positive, chymase-positive, as well as with the simultaneous presence of tryptase and chymase (Fig. 7).

The MC’s hypogranular forms formation reflects the active, unfavorable course of the disease, which is accompanied by a high level of protease synthesis, coupled with active liberation into the extracellular matrix. The formation of immature and mature granules activity can be explained by the stochastic model for MC granule growth and elimination with the direct participation of nano-machines (Hammel and Meilijson 2015). In this case, the homotypic fusion of small granules with each other is possible, leading to the unification of their contents and the outer coating with a common plasma membrane (Fig. 7). At the same time as shown with confocal microscopy, it is possible to suggest the possibility of unequal granules further merging, which might be a common granule maturation and modification mechanism of the secretome intragranular composition (Fig. 6).

Ultimately, the content and composition of the granules reflect the cell physiological state, adequate to the conditions of local homeostasis, which can be significantly distorted in pathology. The dependence of the various sizes in MC granules formation is closely related to the needs of a specific tissue microenvironment in one or another secretome component and an integral secretory activity. As a rule, the size of the granules correlates with the duration of their stay in the mast cell cytoplasm. Small granules have a higher rate of post-translational modification of the proteases enclosed in them, are practically not stored, and are characterized by rapid exchange of secretome components in the course of active secretion into the extracellular matrix. Obviously, in the case of hypogranulated MC in mastocytosis, the specific proteases maturation can be completed at the level of type I and II secretory granules with further active secretion into the extracellular matrix, leading to various clinical or organ-specific manifestations (Figs. 6, 7).

Moreover, in atypical MCs, a certain level of mature secretory granules in the cytoplasm may not be maintained. Further on, when discussing the content of proteases in atypical hypogranulated forms of MC during mastocytosis, one should take into account the fact that young granules may be preferred in secretion over mature ones (Hammel and Meilijson 2015). This fact can serve as an additional explanation for the formation of the hypogranulated MC phenotype in mastocytosis.

### Secretory pathways of specific proteases of MCs in mastocytosis

Evaluation of the secretory pathways, which exert the physiological effects of tryptase and chymase in the intercellular matrix, has a significant information potential. In detail, these processes are described in our previous works (Atiakshin et al. 2018a, 2019). In parallel with the secretome maturation, the molecular nano-machines associated with the granules allow precise regulation of the release of the necessary mediators from the granules with further transportation to the cytoplasm and extracellular matrix (Blank et al. 2014) (Figs. 6, 7). Unfortunately, microscopy does not allow visualization of some events associated with the hypogranulated forms of atypical MC in mastocytosis, such as transgranulation, microvesicular transport, and exosome formation for the secretion of mediators, etc.

Gradual degranulation or a microvesicular transport provide background (constitutive) secretion of tryptase and chymase into the intercellular space, the intensity, despite its weak severity, is determined by the scale of proteases local participation in the local homeostasis regulation (Blank et al. 2014; Vukman et al. 2017) (Fig. 7). However, it is obvious that with the development of mastocytosis, this tryptase and chymase secretion mechanism can acquire significant activity, despite the absence of morphological evidence. MCs are known to have the ability, under certain conditions, to accelerate secretion hundreds of times per unit time (Hammel and Meilijson 2015). Gradual degranulation is an important signaling system for the interaction of MCs with each other. Considering the tight MC neighboring to each other in the red bone marrow, as a pathognomonic sign of the disease, we can assume the active piecemeal degranulation participation in the MC intercellular signaling using specific proteases.

Proteases can be secreted into the tissue microenvironment via the “transgranulation" mechanism, during which micro bulging of MC cytolemma is formed at specific loci in contact with other cells (Fig. 7) (Atiakshin et al. 2018a, 2020). Finally, a visually indetectable mechanism of MC proteases secretion into the extracellular matrix is possible through the exosomes formation (Rabelo Melo et al. 2019) (Fig. 7).

Along with the above degranulation mechanisms, there are other options for proteases release into the extracellular matrix, which was observed in granular forms of atypical MC in mastocytosis, as well as in typical MC of red bone marrow. With the kiss-and-run secretion mechanism (Blank et al. 2014), MC granules came into contact with the plasma membrane to form a temporary pore releasing the proteases into the extracellular matrix in corresponding amount with slightly higher intensity compared to the microvesicular secretion mechanism (Blank et al. 2014) (Fig. 2c, g, h–k, 3a, 7). The peripheral arrangement of granules in type I MC indicates the active use of this mechanism in specific proteases secretion.

### Intragranular localization of specific MC proteases in mastocytosis

The granules structure depends on the degree of maturity, the proteases processing stage and the activity of secretory pathways adequately to the tissue microenvironment challenges. Therefore, in the morphological aspect, it is important to evaluate the topographic features of the arrangement of enzymes in granules. The study of the localization of secretome components in MC granules in mastocytosis was carried out using electron microscopy (Weidner et al. 1992). In the granules, various contrast-rich objects were identified in the form of twisted plates—“scrolls”, as well as grating and/or lattice-like structures, etc. However, these results cannot provide information on the qualitative composition of the visualized structures (Crivellato et al. 2004). In several works with the improved technique of immune-electronic histochemistry, the granules ultrastructure dependence on the presence of proteases was shown; for example, granules with tryptase contained “scrolls” that could overlap each other, and granules with chymase were characterized by the presence of a crystalloid lattice (Dvorak 1995; Shukla et al. 2006; Weidner et al. 1992). Tryptase could be with chymase, carboxypeptidase A and cathepsin G in the same granules (Weidner et al. 1992). However, it should be noted that the solution to the problem of the protease co-localization in granules during electron microscopic examination is very difficult due to the effect of fixing the biomaterial, the histological section plane under study and first, the methodological issues of double immunolabeling.

Our data suggest that contrast-reach formations along the periphery of the granules observed by electron microscopy, as well as possibly scrolls, are a morphological reflection of specific proteases location at these loci (Figs. 5, 6). An immunomorphological study is a very informative addition to electron microscopy, allowing the detection of fluorochrome-labeled proteases in MC, including inside the granules. Modern advances in confocal microscopy with an ultra-high-resolution option provide unique molecular morphological information about the topography of intragranular co-localization of specific MC proteases (Fig. 6).

### Specific MC proteases as multifunctional mediators

MC proteases during the accumulation of granules in the cytoplasm show reduced enzymatic activity due to the characteristics of the pH level and interaction with serglycin and glycosaminoglycans (Hernández-Hernández et al. 2012; Pejler et al. 2010; Ronnberg et al. 2012). In addition to the detection of enzymes using chloroacetate esterase staining technique, an indirect judgment on the content of proteases in MC granules can be obtained after using various metachromatic staining options (Horny 2009; Metcalfe 2008; Sperr et al. 2001; Stevens and Rosenthal 2001).

Specific proteases bio-effects development in the red bone marrow begins from the moment they enter the extracellular matrix and is characterized by a number of specific features. In particular, specific proteases are involved in collagen fibrillogenesis (Atiakshin et al. 2020). This explains the frequent detection of sclerosis and collagen fibrosis in the red bone marrow associated with the prevailing presence of atypical MCs in mastocytosis (Chiu et al. 2009).

Tryptase has a high biological activity, affecting the state of many cellular and non-cellular components of the tissue microenvironment (Caughey 2007, 2011; Hallgren and Pejler 2006; Pejler et al. 2007; Welle 1997). Tryptase has its molecular targets on the cells or components of the extracellular matrix, causing any pro- or anti-inflammatory effects and presumably pro- or anti-oncogenic mechanisms according to other authors (Caughey 2006; Krystel-Whittemore et al. 2015; Ribatti 2016; Chen et al. 2019; Wang et al. 2019; Zhang et al. 2019; Bonadonna et al 2019; Xiao et al. 2019; Rabelo Melo et al. 2019).

The substrates of another specific TK protease, chymase, are various components of the extracellular matrix, receptors, proteins, as well as chemokines and cytokines (Pejler et al. 2007). In addition to the function of angiotensin II as an effector peptide of the renin-angiotensin system, it also has effects on the regulation of cell growth, angiogenesis, regeneration, and tissue remodeling (Atiakshin et al. 2020; Dong et al. 2013). Chymase is capable of causing MC migration to the place of destination in tissues and thus acts as an inducer of directional movement (Zhang et al. 2018). Our previous studies convincingly indicated MCs direct participation in fibrillogenesis using a pronounced inductive effect on the ratio of the tissue microenvironment components, including areas around the fibroblastic differone cell (Atiakshin et al. 2020). Like tryptase, chymase is actively involved in angiogenesis, which contributes to the progression of cancer (Kurihara-Shimomura et al. 2020).

More information about the biological effects of specific mast cell proteases can be found in the relevant reviews (Atiakshin et al. 2018a, b, 2019; Caughey 2011; Pejler et al. 2009).

### Protease profile of MC in the diagnosis of mastocytosis

The ratio of tryptase-positive and chymase-positive MC in the red bone marrow will be of great importance for the specific tissue microenvironment formation, indicating the vector of functional changes presence at a given time. At the same time, it should be noted that the high variability of the expression of the specific protease within each MC, regardless of its type. The co-localization of tryptase and chymase in the same granules (Figs. 6, 7) emphasized the variability of this criterion depending not only on organ affiliation but also on the development of pathology. Thus, the cytological characteristic of the expression and localization of tryptase and chymase in MC is a separate diagnostic value for assessing the progression of mastocytosis.

## Conclusion

The scheme (Fig. 7) shows the main stages of post-translational modification of proteases, taking into account their cytotopography, intragranular localization, and secretory mechanisms. MC proteases are a convenient target for molecular morphological analysis. Detection of nonspecific proteases involved in the MC proteins processing intended for secretion allows interpretation of the activity and intracellular biogenesis features of tryptase and chymase. Intragranular localization and secretory pathways of specific MC proteases depend on the maturity stage of secretory granules. The involvement of specific MC proteases in the mastocytosis pathogenesis makes it worthwhile to further study the tryptase and chymase expression depending on the form of the disease. For diagnosing mastocytosis, it is necessary to determine the MC protease profile of the red bone marrow, taking into account the tryptase-positive and chymase-positive cells ratio and cells with the simultaneous expression of both proteases. Each type of atypical MC in mastocytosis has its cytological characteristics of the expression of the specific protease and, accordingly, its pool volume can make a commensurate contribution during the disease.

MC co-localization with other cells of the red bone marrow is important for myelopoiesis in the red bone marrow and may account for the proteases biological effects in the regulation of local homeostasis and selective morphogenetic processes. Dynamic characteristics of red bone marrow MCs are an additional task of morphological analysis in pathology. A single biopsy gives an idea of the expression of the protease only at a particular moment in the mastocytosis development at the time of taking biomaterial. Understanding the role of protease at each stage of the disease development will expand the diagnostic and therapeutic capabilities not only about the underlying disease but also to concomitant diseases.

Thus, the study of the proteases biological effects in immunomorphological studies expands modern ideas about the MC functional potential, opening up new possibilities in the diagnosis of mastocytosis and therapy monitoring. The MC proteases expression and secretory mechanisms may represent a promising pharmacological target in the mastocytosis treatment.

## Data Availability

All data and materials are available on reasonable request. Address to I.B. (e-mail: buchwalow@pathologie-hh.de) or M.T. (email: mtiemann@hp-hamburg.de) Institute for Hematopathology, Hamburg, Germany.
